# An Overview of the Bacterial Carbonic Anhydrases

**DOI:** 10.3390/metabo7040056

**Published:** 2017-11-11

**Authors:** Claudiu T. Supuran, Clemente Capasso

**Affiliations:** 1 Dipartimento Neurofarba, Sezione di Scienze Farmaceutiche, Laboratorio di Chimica Bioinorganica, Università degli Studi di Firenze, Polo Scientifico, Via U. Schiff 6, Sesto Fiorentino, 50019 Florence, Italy; 2 Istituto di Bioscienze e Biorisorse (IBBR), Consiglio Nazionale delle Ricerche (CNR), via Pietro Castellino 111, 80131 Napoli, Italy

**Keywords:** bacterial carbonic anhydrases, inhibitors, antibiotic, CO_2_ capture, engineered bacteria

## Abstract

Bacteria encode carbonic anhydrases (CAs, EC 4.2.1.1) belonging to three different genetic families, the α-, β-, and γ-classes. By equilibrating CO_2_ and bicarbonate, these metalloenzymes interfere with pH regulation and other crucial physiological processes of these organisms. The detailed investigations of many such enzymes from pathogenic and non-pathogenic bacteria afford the opportunity to design both novel therapeutic agents, as well as biomimetic processes, for example, for CO_2_ capture. Investigation of bacterial CA inhibitors and activators may be relevant for finding antibiotics with a new mechanism of action.

## 1. Introduction

In the time of emerging antibiotic resistance, the improvement of pharmacological arsenal against bacterial pathogens is of pivotal importance [1,2]. Among the strategies adopted for fighting antibiotic resistance, the effectiveness is a structural upgrade of the current clinical drugs for generating novel antibiotics [1,2]. The limit of this strategy is that the newly generated drugs could have a limited lifespan due to the possible resistance that they will develop sooner or later. Fortunately, in the last years, the DNA sequencing approach applied to the bacterial genome allowed the discovery of numerous genes encoding for enzymes which catalyze metabolic pathways essential for the life cycle and/or the virulence of these microbes [3]. Thus, scientists possess in vitro essential bacterial targets for finding and designing new antiinfectives able to disarm pathogens through their inhibition, as well as to bypass their resistance to conventional antimicrobials. In fact, the inhibition of the new bacterial targets takes place through mechanisms different from those usually represented by the block of DNA gyrase, the inhibition of the ribosomal function, and the shut down of the cell-wall biosynthesis, as most clinically used antibiotics act [3]. Moreover, this strategy will result in the development of new antiinfectives, which can replace those already used in clinics with increasing bacterial resistance. In this context, the superfamily of carbonic anhydrases (CAs, EC 4.2.1.1) represents a valuable member of such new macromolecules affecting the growth of microorganisms or making them vulnerable to the host defense mechanisms [4,5,6,7,8]. These metalloenzymes catalyze the simple but physiologically crucial reaction of carbon dioxide hydration to bicarbonate and protons: CO_2_ + H_2_O ⇄ HCO_3_^−^ + H+ [4,8,9,10,11,12,13,14], and they are involved in the transport and supply of CO_2_ or HCO_3_^−^ in pH homeostasis, the secretion of electrolytes, biosynthetic processes, and photosynthesis [15,16]. Moreover, CAs are target molecules of some antibacterial drugs, such as sulfanilamide.

Since CAs are very effective catalysts for the conversion of CO_2_ to bicarbonate, the CA superfamily might be involved in the capture/sequestration of CO_2_ from combustion gases with the goal of alleviating the global warming effects through a reduction of CO_2_ emissions in the atmosphere [17]. The production of CO_2_ is linked to the industrial development that must necessarily reduce its production. To decrease the amount of CO_2_ in the atmosphere, a number of CO_2_ sequestration methods have been proposed [17]. Most of them require that the CO_2_ captured from the flue gases is compressed, transported to the sequestration site, and injected into specific areas for long-term storage [17]. All these procedures lead to an increase in the costs of the capture and storage processes [17]. For this reason, the biomimetic approach represents a valid strategy for CO_2_ capture. It allows the CO_2_ conversion to water-soluble ions and offers many advantages over other methods, such as its eco-compatibility and the possibility to use the reaction products for multiple applications, with no added costs. Furthermore, thermophilic CAs are still active at high temperatures compared to their mesophilic counterparts, and their use is preferred in environments characterized by hard conditions, such as those of the carbon capture process (high temperature, high salinity, extreme pH) [18,19,20,21].

## 2. Classification and Structure

### 2.1. CA-Classes

**Figure 1:**
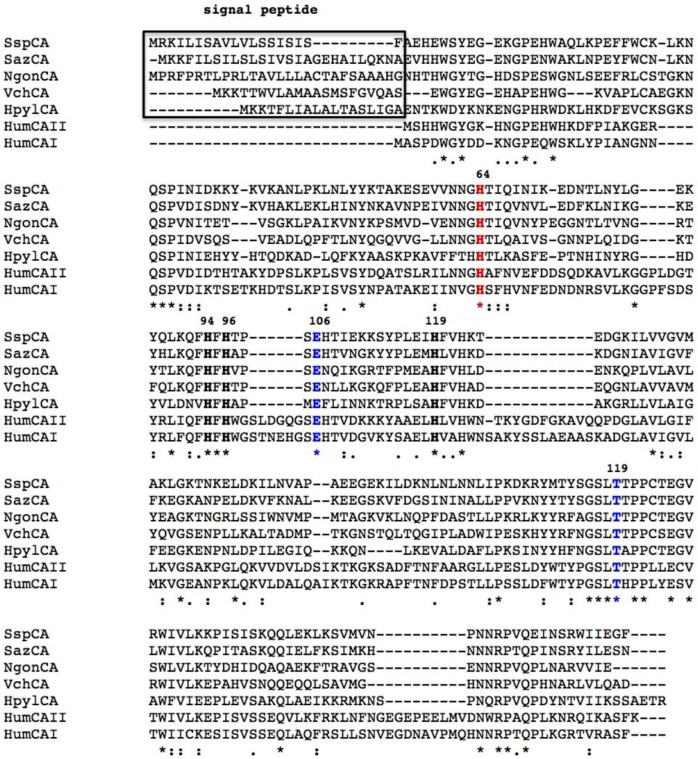
Multi-alignment of the amino acid sequences of two human α-CAs (hCAI and hCAII) and of five bacterial α-CAs (SspCA, SazCA, NgoCA, VchCA, and HypyCA) was performed with the ClustalW program, version 2.1. The hCA I numbering system was used. Black bold indicates the amino acid residues of the catalytic triad; blue bold represents the “gate-keeper” residues; and red bold shows the “proton shuttle residue”. Box indicates the signal peptide. The asterisk (*) indicates identity at a position; the symbol (:) designates conserved substitutions, while (.) indicates semi-conserved substitutions. Multi-alignment was performed with the program Clustal W, version 2.1. Legend: hCAI, α-CA isoform I from *Homo sapiens*; hCAII, α-CA isoform II from *Homo sapiens*; SspCA, α-CA from *Sulfurihydrogenibium yellowstonense;* SazCA*,* α-CA from *Sulfurihydrogenibium azorense;* NgonCA, α-CA from *Neisseria gonorrhea*; VchCA, α-CA from *Vibrio cholerae*; HpyCA, α-CA from *Helicobacter pylori*.

**Figure 2:**
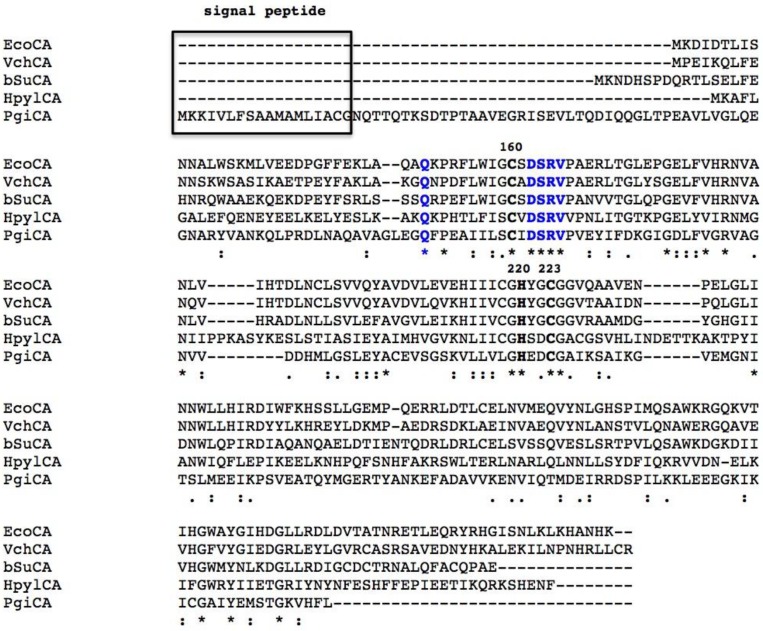
Alignment of the amino acid sequences of bacterial 𝛽-CAs from different species. Zinc ligands are indicated in black bold; amino acids involved in the enzyme catalytic cycle are indicated in blue bold. Box indicates the signal peptide. The asterisk (*) indicates identity at a position; the symbol (:) designates conserved substitutions, while (.) indicates semi-conserved substitutions. Multi-alignment was performed with the program Clustal W, version 2.1. *Pisum sativum* numbering system was used. Legend: EcoCA, 𝛽-CA from *Escherichia coli*; VchCA, 𝛽-CA from *Vibrio cholerae*; bSuCA, 𝛽-CA from *Brucella suis*; HpyCA, 𝛽-CA from *Helicobacter pylori*; PgiCA, 𝛽-CA from *Porphyromonas gingivalis*.

**Figure 3:**
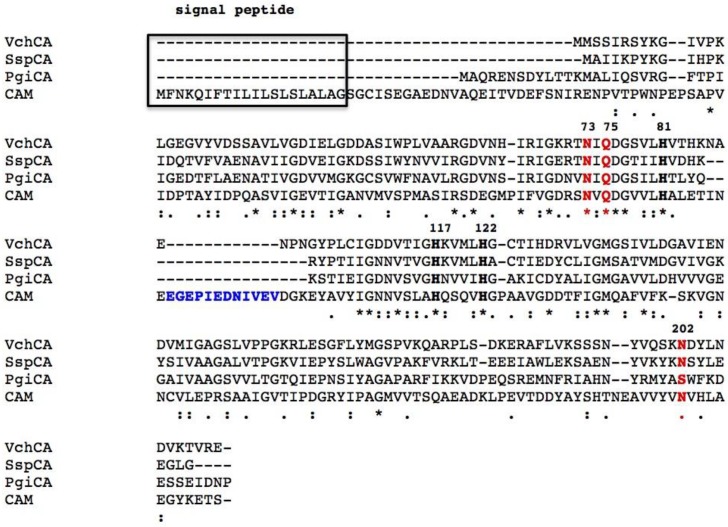
Amino acid sequence alignment of the 𝛾-CAs from different bacterial sources, such as *Vibrio cholerae*, *Sulfurihydrogenibium yellowstonense, Porphyromonas gingivalis*, and *Methanosarcina thermophila*. The metal ion ligands (His81, His117, and His122) are indicated in black bold; the catalytically relevant residues of CAM, such as Asn73, Gln75, and Asn202, which participate in a network of hydrogen bonds with the catalytic water molecule, are indicated in red bold; the acidic loop residues containing the proton shuttle residues (Glu89) are colored in blue bold, but are missing in PgiCA. The CAM numbering system was used. Box indicates the signal peptide. Legend: VchCA (𝛾-CA from *Vibrio cholerae*), SspCA (𝛾-CA from *Sulfurihydrogenibium yellowstonense*), PgiCA (𝛾-CA from *Porphyromonas gingivalis*), and CAM (𝛾-CA from *Methanosarcina thermophila*). The asterisk (*) indicates identity at all aligned positions; the symbol (:) relates to conserved substitutions, while (.) means that semi-conserved substitutions are observed. The multi-alignment was performed with the program Clustal W.

The CAs make up a widely distributed class of metalloenzymes with the catalytically active species represented by a metal hydroxide derivative [4,5,6,7,8]. CAs are grouped into seven genetically distinct families, named α-, β-, γ-, δ-, ζ-, η-, and ɵ-CAs, with different folds and structures but common CO_2_ hydratase activity, coupled to low sequence similarity. Bacteria encode for enzymes belonging to α-, β-, and γ-CA classes [8,22,23,24,25,26,27]. Bacteria have a very intricate CA distribution pattern because some of them encode CAs belonging to only one family, whilst others encode those from two or even three different genetic families. The α- and β-CAs are metalloenzymes, which use the Zn(II) ion as a catalytic metal; γ-CAs are Fe(II) enzymes, but they are also active with bound Zn(II) or Co(II) ions [28,29,30,31,32,33,34,35]. The metal ion from the CA active site is coordinated by three His residues in the α- and γ-classes (Figure 1 and Figure 2), and by one His and two Cys residues in the β-class (Figure 3). The fourth ligand is a water molecule/hydroxide ion acting as a nucleophile in the catalytic cycle of the enzyme [8,24,25,36,37,38,39]. The rate-determining step of the entire catalytic process is the formation of the metal hydroxide species of the enzyme by the transfer of a proton from the metal-coordinated water molecule to the surrounding solvent, possibly via proton-shuttling residues [5,8,22,24,25].

### 2.2. α-CA Structure

**Figure 4:**
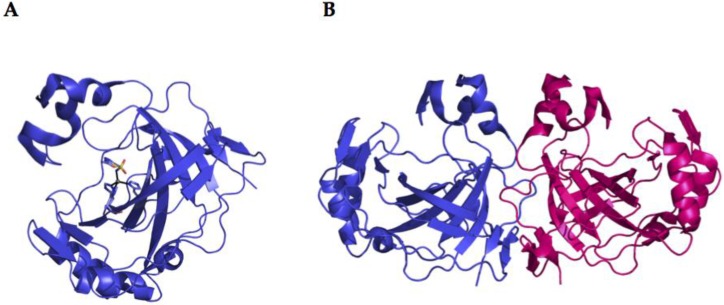
Ribbon representation of the overall fold of α-CA (SspCA) from *Sulfurihydrogenibium yellowstonense*. (**A**): SspCA active monomer with the inhibitor acetazolamide (**AAZ**) showed; (**B**): SspCA active dimer.

Bacterial *α-CAs* have only been poorly characterized with respect to the mammalian *α-*CAs. In fact, the CAs from *Neisseria gonorrhoeae*, *Sulfurihydrogenibium yellowstonense, Sulfurihydrogenibium azorense*, and *Thermovibrio ammonificans* are the only bacterial α-CAs with a known three-dimensional structure [30,33,40,41]. An example of the typical structural organization of a bacterial α-CA is offered by the X-ray crystal structure of the CA identified in the thermophilic bacterium *Sulfurihydrogenibium yellowstonense* YO3AOP1 (Figure 4) [30,33]. This three-dimensional structure generally resembles those of human α-CAs and it was obtained in the presence of the classical inhibitor of CAs, the sulfonamide acetazolamide (AAZ). In particular, it shows a homodimeric arrangement stabilized by a large number of hydrogen bonds and several hydrophobic interactions. The crystallized α-CAs are active as monomers and dimers (Figure 4). The active site is located in a deep cavity, which extends from the protein surface to the center of the molecule, and is characterized by hydrophilic and hydrophobic regions. The hydrophilic part assists in the transfer of the proton from the Zn-bound water to the solvent, while the hydrophobic district is involved in CO_2_ binding and ligand recognition. The catalytic zinc ion is located at the bottom of this cavity and is tetrahedrally coordinated by three histidine residues and by the N atom of the sulfonamide moiety of the inhibitor (or probably by the water molecule in the uninhibited enzyme). Intriguingly, the bacterial α-CAs show a more compact structure with respect to the mammalian counterpart, which is characterized by the presence of three insertions (Figure 1) [30,33]. Due to the absence of these inserts, an active site larger than that of human enzymes characterizes the bacterial CAs. Moreover, the structure of the thermostable CAs, such as SspCA (from *Sulfurihydrogenibium yellowstonense)* and SazCA (from *Sulfurihydrogenibium azorense*) identified in thermophilic bacteria, are characterized by a higher content of secondary-structural elements and an increased number of charged residues, which are all elements responsible for protein thermostability [30,33]. It is interesting to note that the crystal structure of TaCA from *Thermovibrio ammonificans* is tetrameric, with a central core stabilized by two intersubunit disulfides and a single lysine residue from each monomer, which is involved in intersubunit ionic interactions [40].

### 2.3. β-CA Structure

**Figure 5:**
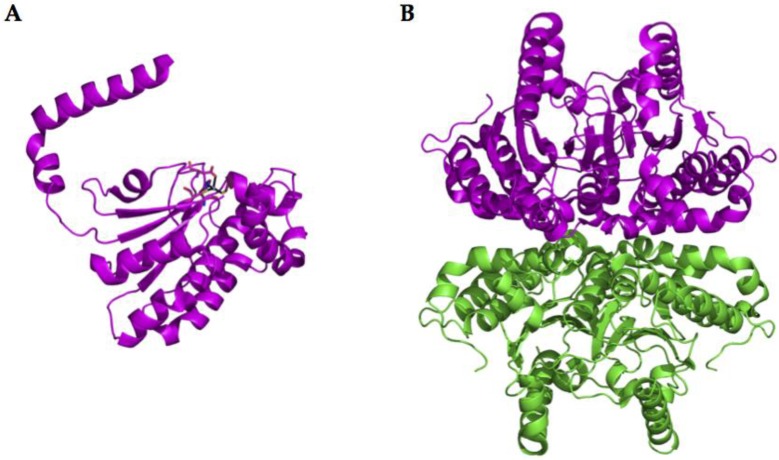
Ribbon representation of the catalytically inactive monomer (**A**) and active tetramer (**B**) of 𝛽-CA (VchCA) from *Vibrio cholerae.*

X-ray crystal structures are available for several of *β-CAs*, such as those from *Escherichia coli*, *Haemophilus influenzae*, *Mycobacterium tuberculosis*, *Salmonella enterica*, and *Vibrio cholerae* [29,42,43,44,45]. The 3-D folds of these enzymes are rather conserved, although some of them are dimers whereas others are tetramers. All bacterial β-CAs crystallized so far are active as dimers or tetramers, with two or four identical active sites. Their shape is that of a rather long channel at the bottom of which the catalytic zinc ion is found, tetrahedrally coordinated by two cysteines, one-histidine and one-aspartic amino acid residue (the so called “closed active site”). Interesting, the enzyme structure from *Vibrio cholerae* (VchCAβ) was determined in its closed active site form at pH values <8.3 (Figure 5) [29]. The “closed active site” is named in this way as these enzymes are not catalytically active (at pH values <8.3). Interesting, in its inactive form, the bicarbonate is bound in a pocket close to the zinc ion [29]. However, at pH values >8.3, the “closed active site” is converted to the “open active site” (with gain of catalytic activity), which is associated with a movement of the Asp residue from the catalytic Zn(II) ion, with the concomitant coordination of an incoming water molecule approaching the metal ion [29]. This water molecule (as hydroxide ion) is, in fact, responsible for the catalytic activity, as shown above for the α-CAs.

### 2.4. γ-CA Structure

**Figure 6:**
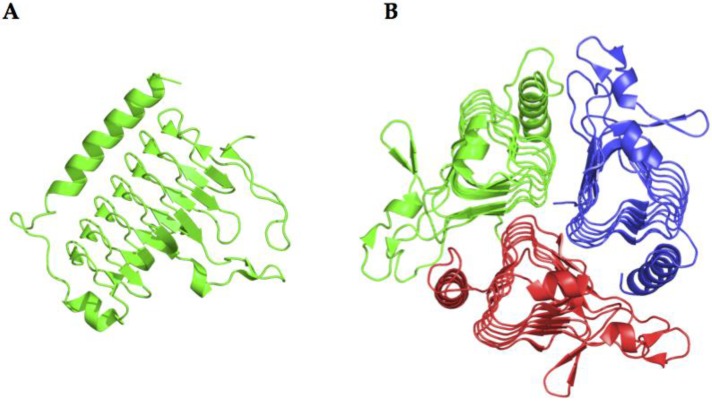
Structural representation of the catalytically inactive monomer (**A**) and active trimer (**B**) of the CAM (γ-CA) enzyme from *Methanosarcina thermophila*.

CAM (Carbonic Anhydrase Methanosarcina) from *Methanosarcina thermophila* is the prototype of the γ-class carbonic anhydrase and the only enzyme from this class that has been crystallized so far (Figure 6) [46]. This enzyme adopts a left-handed parallel β-helix fold and crystallizes as a trimer with three zinc-containing active sites, each located at the interface between two monomers. The metalloenzyme is only active as a trimer (Figure 6) [46]. Interestingly, in this class of enzyme, instead of a histidine (as in α-CAs), there is a glutamic acid residue acting as a proton shuttle residue (Figure 3). In fact, the high-resolution crystal of CAM showed that Glu89 has two orientations, similar to those of His64 in α-CAs (Figure 3) [46].

## 3. Catalytic Activity

**Figure 7:**
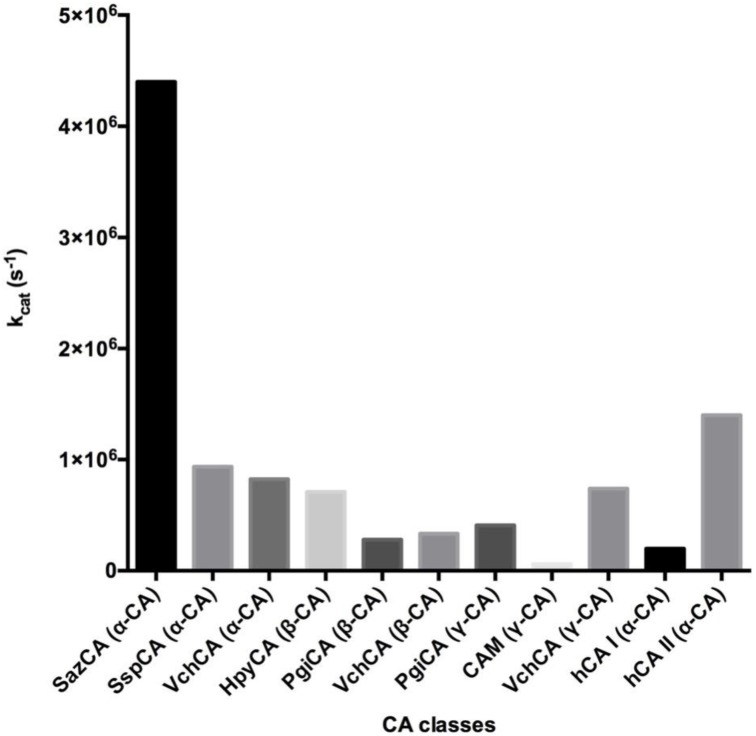
Kinetic parameters for the CO_2_ hydration reaction catalyzed by the human cytosolic isozymes hCA I and II (α-class CAs) and bacterial α-, β-, and γ-CAs, such as SazCA (α-CAs from *Sulfurihydrogenibium azorense*), SspCA (α-CAs from *Sulfurihydrogenibium yellowstonense)*, HpyCA (α- and β-CAs from *Helicobacter pylori*), VchCA (α-, β-, and γ-CAs from *Vibrio cholerae*), PgiCA (β- and γ-CAs from *Porphyromonas gingivalis*), and CAM (γ-CA from *Methanosarcina thermophila*). All the measurements were done at 20 °C, pH 7.5 (α-class enzymes), and pH 8.3 (β- and γ-CAs) by a stopped flow CO_2_ hydratase assay method.

The spontaneous reversible CO_2_ hydration reaction in the absence of the catalyst has an effective first-order rate constant of 0.15 s^−1^, while the reverse reaction shows a rate constant of 50 s^−1^ [36,47]. In the living organisms, the CO_2_ hydration and the HCO_3_^−^ dehydration are connected to very fast processes, such as those related to transport/secretory processes. The main metabolic role of CAs is to catalyze the carbon dioxide hydration at a very high rate, with a pseudo first order kinetic constant (k_cat_) ranging from 10^4^ to 10^6^ s^−1^ [36,47]. Thus, the CA superfamily significantly accelerates the hydration reaction to support the metabolic processes involving dissolved inorganic carbon. Until 2012, the most active CA was the human isoform hCA II (k_cat_ = 1.40 × 10^6^ s^−1^), belonging to the α-class and abundantly present in the human erythrocytes [36,48]. The hCA II, at the level of the peripheral tissues, converts the CO_2_ into carbonic acid, while when the blood reaches the lungs, dehydrates the HCO_3_^−^ to CO_2_ for it exhalation. In 2012, a new α-CA was identified, and was shown to be a highly and catalytically effective catalyst for the CO_2_ hydration reaction (Figure 7) [49]. To our surprise, this CA (SazCA) was identified in the genome of the thermophilic bacterium *Sulfurihydrogenibium azorense* and showed a k_cat_ = 4.40 × 10^6^ s^−1^, thus being 2.33 times more active than the human isoform hCA II (Figure 7) [30,49]. In general, the bacterial CAs belonging to the three known classes (α, β, and γ) are efficient catalysts for the CO_2_ hydration reaction. Analyzing the three-dimensional structures of the bacterial CAs, it has been observed that the catalytic pocket is rather small for the γ-CAs, gets bigger for β-CAs, and becomes quite large in the α-CAs (Figure 4, Figure 5 and Figure 6) [5,8,25,50]. As a consequence, the catalytic constant of the γ-CAs is usually low compared to the β-CAs, which is lower when compared to many bacterial α-CAs (Figure 7). Sometimes, there are γ-CAs with a catalytic turnover number that is higher with respect to that shown by the β-class, such as the γ-CAs from *Porphyromonas gingivalis* and *Vibrio cholerae* (Figure 7).

## 4. CA Inhibitors

**Figure 8:**
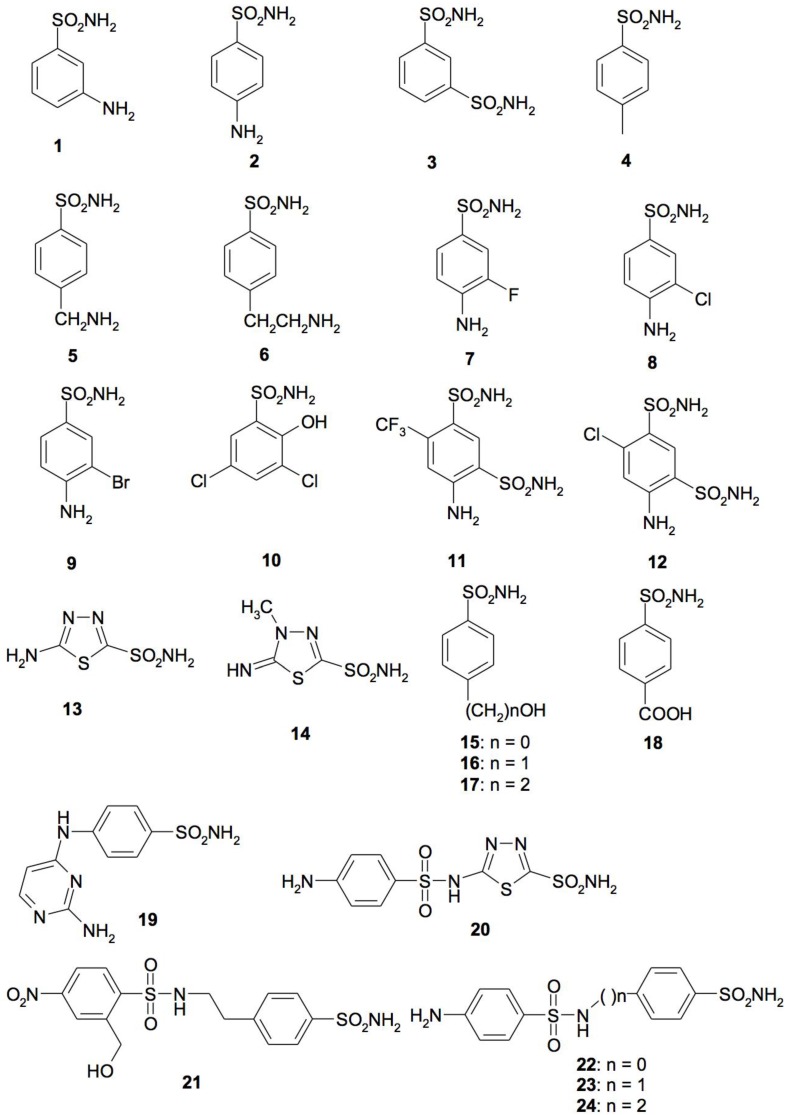

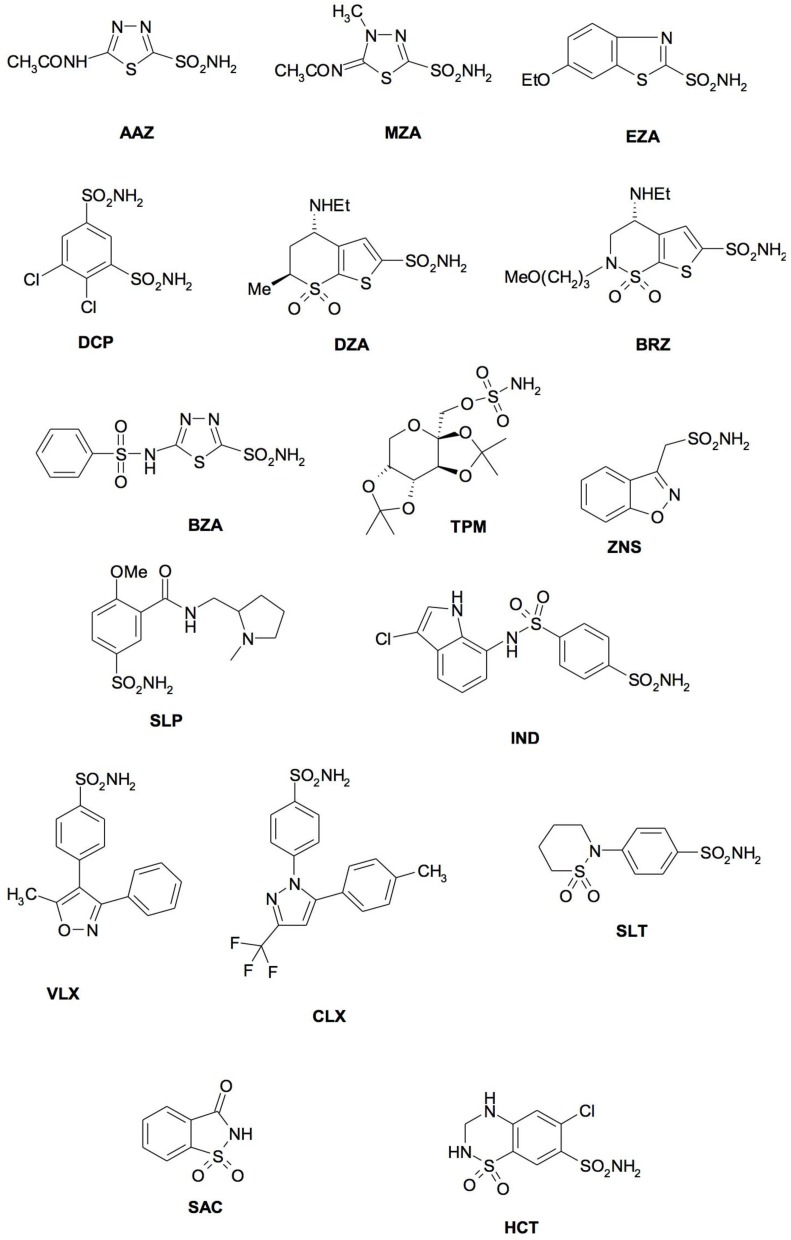
Sulfonamides, sulfamates, and some of their derivatives investigated as bacterial CA inhibitors.

Different types of CA inhibitors (CAIs) exist [47,48] and they can be grouped into: (1) the metal ion binders (anion, sulfonamides, and their bioisosteres, dithiocarbamates, xanthates, etc.); (2) compounds which anchor to the zinc-coordinated water molecule/hydroxide ion (phenols, polyamines, thioxocoumarins, sulfocumarins); (3) compounds occluding the active site entrance, such as coumarins and their isosteres; and (4) compounds binding out of the active site [47]. This subdivision has been made considering the way that the inhibitors bind the catalytic metal ion, the metal coordinated-water molecule, and the occlusion of the active site [47]. The most investigated CAIs are anions and sulfonamides [36,47,51,52]. Sulfonamides were discovered by Domagk in 1935 [53], and were the first antimicrobial drugs. The first sulfonamide showing effective antibacterial activity was Prontosil, a sulfanilamide prodrug isosteric/isostructural with p-aminobenzoic acid (PABA) [54]. In the following years, a range of analogs constituting the so-called sulfa drug class of anti-bacterials entered into clinical use, and many of these compounds are still widely used. A library of 40 compounds, 39 sulfonamides, and one sulfamate was used to provide CAIs (Figure 8) [6,10,13,14,55,56,57,58,59,60,61,62,63,64,65,66].

Derivatives **1–24** and **AAZ-HCT** are either simple aromatic/heterocyclic sulfonamides widely used as building blocks for obtaining new families of such pharmacological agents, or they are clinically used agents, among which acetazolamide (**AAZ**), methazolamide (**MZA**), ethoxzolamide (**EZA**), and dichlorophenamide (**DCP**) are the classical, systemically acting antiglaucoma CAIs. Dorzolamide (**DZA**) and brinzolamide (**BRZ**) are topically acting antiglaucoma agents; benzolamide (**BZA**) is an orphan drug belonging to this class of pharmacological agents. Moreover, the zonisamide (**ZNS**), sulthiame (**SLT**), and the sulfamic acid ester topiramate (**TPM**) are widely used antiepileptic drugs. Sulpiride (**SLP**) and indisulam (**IND**) were also shown by our group to belong to this class of pharmacological agents, together with the COX2 selective inhibitors celecoxib (**CLX**) and valdecoxib (**VLX**). Saccharin (**SAC**) and the diuretic hydrochlorothiazide (**HCT**) are also known to act as CAIs. Sulfonamides, such as the clinically used derivatives acetazolamide, methazolamide, ethoxzolamide, dichlorophenamide, dorzolamide, and brinzolamide, bind in a tetrahedral geometry to the Zn(II) ion in the deprotonated state, with the nitrogen atom of the sulfonamide moiety coordinated to Zn(II) and an extended network of hydrogen bonds, involving amino acid residues of the enzyme, also participating in the anchoring of the inhibitor molecule to the metal ion [36,47,48,67]. The aromatic/heterocyclic part of the inhibitor interacts with the hydrophilic and hydrophobic residues of the catalytic cavity [36,47,51,52].

Anions, such as inorganic metal-complexing anions or more complicated species such as carboxylates, are also known to bind to CAs [47,48]. These anions may bind either the tetrahedral geometry of the metal ion or as trigonal–bipyramidal adducts. Anion inhibitors are important both for understanding the inhibition/catalytic mechanisms of these enzymes fundamental for many physiologic processes, and for designing novel types of inhibitors which may have clinical applications for the management of a variety of disorders in which CAs are involved [47,48].

In the last ten years, numerous results concerning the inhibition profile of the three bacterial CA classes (α, β, and γ) have been reported using anions and sulfonamides. Most of these studies were carried out on bacterial CAs from pathogenic bacteria, such as *Francisella tularensis*, *Burkholderia pseudomallei*, *Vibrio cholerae*, *Streptococcus mutans*, *Porphyromonas gingivalis*, *Legionella pneumophila*, *Clostridium perfringens*, and *Mycobacterium turberculosis*, etc. [6,14,68,69,70]. The results indicated that certain CAIs were able to highly inhibit most of the CAs identified in the genome of the aforementioned bacteria (for details see associate bibliography) [4,62,71,72,73,74]. Moreover, certain CAIs, such as acetazolamide and methazolamide, were shown to effectively inhibit bacterial growth in cell cultures [75]. The inhibition profile with simple and complex anions, as well as small molecules inhibiting other CAs, showed that the most efficient inhibitors detected so far are sulfamide, sulfamate, phenylboronic acid, and phenylarsonic acid [24,62,76]. Generally, halides, cyanide, bicarbonate, nitrite, selenate, diphosphate, divanadate, tetraborate, peroxodisulfate, hexafluorophosphate, and triflate exhibit weak inhibitory activity against the bacterial CAs [22,24,25,76,77].

## 5. Activators

**Figure 9:**
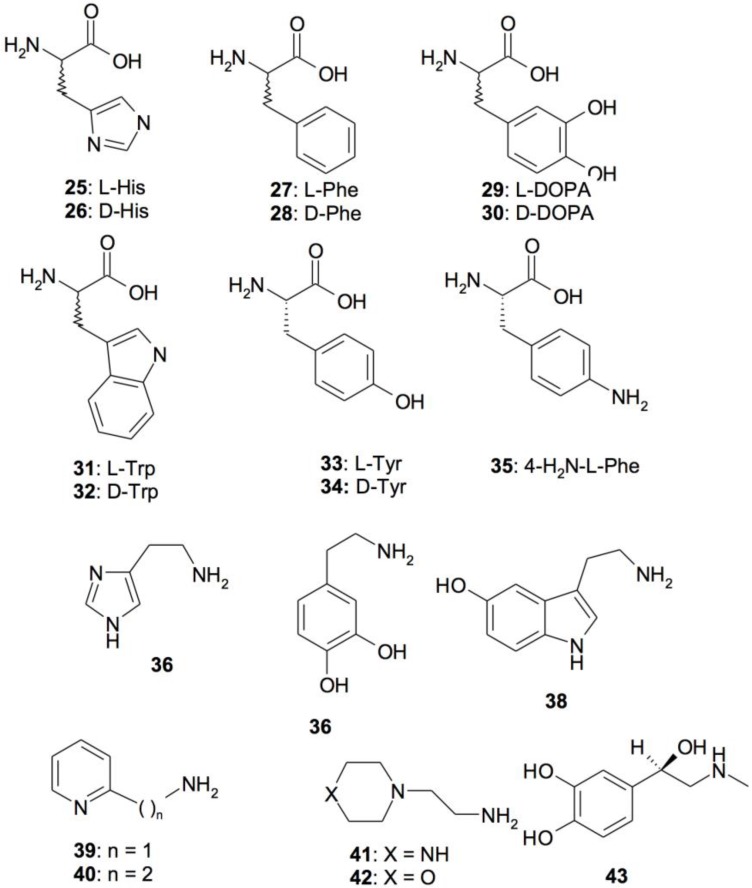
Amino acid and amine CA activators **25**–**43** investigated for their interaction with bacterial CAs.

**Figure 10:**
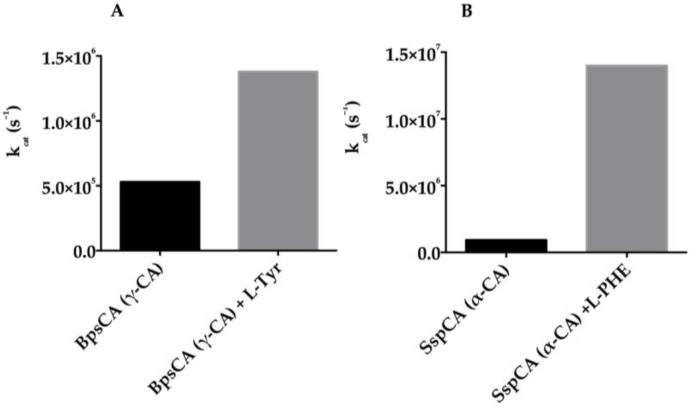
(**A**): Activation of the bacterial BpsCA (γ-CA) with L-Tyr; (**B**): Activation of the bacterial SspCA (α-CA) L-Phe. All measurements were carried out at 25 °C and pH 7.5, for the CO_2_ hydration reaction.

An interesting feature of the CA superfamily is that they can bind within the middle-exit part of the active site molecules known as “activators” (CAA). They are biogenic amines (histamine, serotonin, and catecholamines), amino acids, oligopeptides, or small proteins (Figure 9 shows the small molecule CAAs mostly investigated) [78,79,80,81]. By means of electronic spectroscopy, X-ray crystallography, and kinetic measurements, it has been demonstrated that CAAs do not influence the binding of CO_2_ to the CA active site but mediate the rate-determining step of the catalysis hurrying the transfer of protons from the active site to the environment. The final result is an overall increase of the catalytic turnover. Thus, the CA activators enhance the k_cat_ of the enzyme, with no effect on KM [78,80,81]. Numerous studies concerning the activation of the mammalian enzymes with amines and amino acids are reported in the literature [78,80,81]. In fact, CAAs may have pharmacologic applications in therapy memory, neurodegenerative diseases (Alzheimer’s disease), or the treatment of genetic CA deficiency syndromes [78,80,81]. On the other hand, the activation of CA classes different from those belonging to mammals has been poorly investigated. Considering the limited data available at this moment on the activation of other classes of CAs and using a series of structurally related amino acids and amines of types **25**–**43** (Figure 9), Supuran and coworkers have investigated the activation profiles of some bacterial CAs [82,83]. More precisely, the activation profile of the γ-CA (BpsCA) identified in the genome of the pathogenic bacteria *Burkholderia pseudomallei* has been investigated for understanding the role of the CAs in the lifecycle and virulence of these bacteria [82]. Moreover, the activation profile of the thermophilic α-CAs (SspCA, from *Sulfurihydrogenibium yellowstonense* and SazCA, from *Sulfurihydrogenibium azorense*) has also been explored [83]. From Figure 10, it is readily apparent that the activators L-Tyr for BpsCA and L-Phe for SspCA enhanced the values of the k_cat_ by one order of magnitude compared to those without activators.

## 6. Phylogenetic Analysis

The complex distribution of the various CA classes in Gram-positive and -negative bacteria allowed us to find a correlation between the evolutionary history of the bacteria and the three CA classes (α, β, and γ) identified in their genome. Prokaryotes appeared on the Earth 3.5–3.8 billion years ago, while eukaryotes were dated to 1.8 billion years ago [84]. During the first 2.0–2.5 billion years, the Earth's atmosphere did not contain oxygen, and the first organisms were thus anaerobic. Eukaryotic organisms’ almost aerobes developed on the Earth when the atmosphere was characterized by a stable and relatively high oxygen content [84]. The oldest part of the evolutionary history of the planet and more than 90% of the phylogenetic diversity of life can be attributed to the microbial world. Moreover, the fact that the Archaea are distinct from other prokaryotes is demonstrated by the existence of protein sequences that are present in Archaea, but not in eubacteria [85]. Many phylogenetic methods support a close correlation of Archaea with Gram-positive bacteria, while Gram-negative bacteria form a separate clade, indicating their phylogenetic distinction. Gupta et al. believe that the Gram-positive bacteria occupy an intermediate position between Archaea and Gram-negative bacteria, and that they evolved precisely from Archaea [25,77]. Phylogenetic analysis of carbonic anhydrases identified bacteria Gram-positive and negatively showed that the ancestral CA is represented by the γ-class. In fact, the γ-CA is the only CA class, which has been identified in Archaea [86,87,88,89]. This is consistent with the theory that maintains a close relationship between the Archaea and the Gram-positive bacteria, considering that Gram-negative arised from the latter. Furthermore, phylogenetic analysis of bacterial CAs showed that the α-CAs, exclusively present in Gram-negative bacteria, were the most recent CAs. These results have been corroborated by the enzymatic promiscuity theory, which is the ability of an enzyme to catalyze a side reaction in addition to the main reaction [90,91]. In fact, as reported in the literature, the α-CAs can catalyze a secondary reaction, such as the hydrolysis of p-NpA or a thioester, in addition to the primary reaction consisting of CO_2_ hydration.

## 7. Localization and Physiological Role

A common feature of all bacterial *α-*CAs known to date is the presence of an N-terminal signal peptide, which suggests a periplasmic or extracellular location (Figure 1). From these findings, we have speculated that in Gram-negative bacteria, the α-CA are able to convert the CO_2_ to bicarbonate diffused in the periplasmic space ensuring the survival and/or satisfying the metabolic needs of the microorganism [25,77]. In fact, several essential metabolic pathways require either CO_2_ or bicarbonate as a substrate, and probably, the spontaneous diffusion of CO_2_ to the outer membrane and the conversion to bicarbonate inside the cell are not sufficient for the metabolic needs of the microorganism. On the contrary, β- or γ-classes have a cytoplasmic localization and are responsible for CO_2_ supply for carboxylase enzymes, pH homeostasis, and other intracellular functions [25,77]. Not all the Gram-negative bacteria, however, have α-CAs. Probably, the α-CAs are not required when the Gram-negative bacteria colonize habitats defined as not metabolic limiting or adverse to their survival [77]. Recently, we analyzed the amino acid sequence of the β-CAs encoded by the genome of Gram-negative bacteria with SignalP version 4.1, which is a program designed to discriminate between signal peptides and transmembrane regions of proteins. The program is available as a web tool at http://www.cbs.dtu.dk/services/SignalP/ [92]. We noted that the primary structure of some β-CAs identified in the genome of some pathogenic Gram-negative bacteria, such as such as HpyCA (from *Helicobacter pylori*), VchCA (from *Vibrio cholerae*), NgonCA (from *Neisseria gonorrhoeae*), and SsalCA (from *Streptococcus salivarius*)*,* present a pre-sequence of 18 or more amino acid residues at the N-terminal part, which resulted in a secretory signal peptide [25,77]. Intriguingly, during the writing of this review, we saw that the CAM enzyme also contained a short putative signal peptide at its N-terminus (Figure 3). Since the signal peptide is essential for the translocation across the cytoplasmic membrane in prokaryotes, it has been suggested that the β- and/or γ-CAs found in Gram-negative bacteria and characterized by the presence of a signal peptide might exhibit a periplasmic localization and a role similar to that described previously for the *α-*CAs [25,77]. 

In the past ten years, the understanding of the function of the bacterial CAs has increased significantly [25,77]. We suggested that the activity of CAs is connected with the survival of the microbes because the metabolic reaction catalyzed by CA is essential for supporting numerous physiological functions involving dissolved inorganic carbon. For example, in non-pathogenic bacteria such as *Ralstonia eutropha* (Gram-negative bacterium found in soil and water) and *Escherichia coli* (a facultative Gram-negative bacterium), it has been demonstrated in vivo that the bacterial growth at an ambient CO_2_ concentration is dependent on CA activity [93,94]. In fact, the CO_2_ and bicarbonate are both produced and consumed by bacterial metabolism. Since CO_2_ is rapidly lost from the bacterial cells by passive diffusion, their rate is maintained individually in balance by the CA activity. In fact, the reversible spontaneous CO_2_ hydratase reaction is insufficient to restore the amount of dissolved inorganic carbon. More interesting is the in vivo evidence concerning the involvement of CAs for the growth of pathogenic bacteria. For example, CAs encoded by the genome of *Helicobacter pylori,* a Gram-negative, microaerophilic bacterium colonizing the human stomach, are essential for the acid acclimatization of the pathogen within the stomach and thus, for bacterial survival in the host [15,16,95,96]. In the case of the pathogenic bacterium *Vibrio cholera* (Gram-negative bacterium responsible of cholera), its CAs are involved in the production of sodium bicarbonate, which induces cholera toxin expression [15,24,61,95,96,97,98,99,100]. Probably, *V. cholera* uses the CAs as a system to colonize the host [6,12,14,101]. Again, the causative agent of brucellosis *Brucella suis*, a non-motile Gram-negative coccobacillus, and the *Mycobacterium tuberculosis*, an obligate pathogenic bacterium responsible for tuberculosis, are needed for the growth of functional CAs [66,102,103,104].

## 8. Engineered Bacteria with a Thermostable CA for CO_2_ Capture

Recently, the heterologous expression of the recombinant thermostable SspCA by the high-density fermentation of *Escherichia coli* cultures, in order to produce a usable biocatalyst for CO_2_ capture, has been described [20]. The enzyme was covalently immobilized onto the surface of magnetic Fe_3_O_4_ nanoparticles (MNP) by using the carbodiimide activation reaction [20]. This approach offered two main advantages: 1) the magnetic nanoparticles-immobilized SspCA via carbodiimide increased the stability and the long-term storage of the biocatalyst; and 2) the immobilized biocatalyst can be recovered and reused from the reaction mixture by simply applying a magnet or an electromagnet field because of the strong ferromagnetic properties of Fe_3_O_4_ [20]. The main issues of this method are the costs connected to biocatalyst purification and the support used for enzyme immobilization. Often, all these aspects may discourage the utilization of enzymes in industrial applications. In 2017, a system able to overexpress and immobilize the protein directly on the outer membrane of *Escherichia coli* for lowering the costs of the purification of the biocatalyst and immobilization has been proposed [105]. To accomplish this, the *Escherichia coli* cells have been engineered using the well-described INP (Ice Nucleation Protein) technique [105]. Briefly, an expression vector composed of a chimeric gene resulting from the fusion of a signal peptide, the *Pseudomonas syringae* INP domain (INPN), and the SspCA gene encoding for the thermostable α-CA, SspCA, has been prepared. During protein overexpression, the signal peptide makes possible the translocation of the neo-synthetized protein through the cytoplasmic membrane, while the INPN domain is necessary for guiding and anchoring the protein to the bacterial outer membrane. The results demonstrated that the anchored SspCA was efficiently overexpressed and active on the bacterial surface of *E. coli* [105]. Moreover, the anchored SspCA was stable and active for 15 h at 70 °C and for days at 25 °C [105]. This approach with respect to the covalent immobilization of the enzyme onto the surface of magnetic Fe_3_O_4_ nanoparticles (MNP) clearly has important advantages. It is a one-step procedure for overexpressing and immobilizing the enzyme simultaneously on the outer membrane, and it drastically reduces the costs needed for enzyme purification, enzyme immobilization, and the support necessary for biocatalyst immobilization [105]. In addition, the biocatalyst could be recovered by a simple centrifugation step from the reaction mixture. The strategy of the INPN-SspCA obtained by engineering *E. coli* could be considered as a good method for approaching the biomimetic capture of CO_2_ and other biotechnological applications in which a highly effective, thermostable catalyst is needed.

## 9. Conclusions

Bacterial CAs were rather poorly investigated until recently. However, in the last years the cloning, purification, and characterization of many representatives, belonging to all three genetic families present in Bacteria, has led to crucial advances in the field. The role of CAs in many pathogenic as well as non-pathogenic bacteria is thus beginning to be better understood. Apart from pH regulation and adaptation to various niches in which bacteria live (e.g., the highly acidic environment in the stomach, in the case of *Helicobacter pylori*, the alkaline one in the gut for *Vibrio cholerae*, etc.), CAs probably participate in biosynthetic processes in which bicarbonate or CO_2_ are substrates, as in the case of other organisms for which these roles are demonstrated. The inhibition and activation of bacterial CAs may be exploited either from pharmacological or environmental viewpoints. On the one hand, the inhibitors of such enzymes may lead to antibiotics with a new mechanism of action, devoid of the drug resistance problems encountered with the various classes of clinically used agents. Moreover, catalytically highly efficient, thermally stable bacterial CAs may have interesting applications for biomimetic CO_2_ capture in the context of global warming due to the accumulation of this gas in the atmosphere as a consequence of anthropic activities. Furthermore, Ca activators of such enzymes may represent an even more attractive option for mitigating global warming.
